# Femoral neck fractures in adults with emphasis on surgical treatment

**DOI:** 10.2340/17453674.2025.44354

**Published:** 2025-08-18

**Authors:** Cecilia ROGMARK, Bjarke VIBERG, Olof WOLF, Sebastian MUKKA, Matthew L COSTA, Jan-Erik GJERTSEN

**Affiliations:** 1 Department of Orthopaedics, Lund University, Skåne University Hospital, Malmö, Sweden; 2 Department of Orthopaedic Surgery and Traumatology, Odense University Hospital, Odense, Denmark; 3 Department of Surgical Sciences, Orthopaedics, Uppsala University, Uppsala, Sweden; 4 Department of Diagnostics and Intervention (Orthopaedics), Umeå University, Umeå, Sweden; 5 Oxford Trauma & Emergency Care, Nuffield Department of Orthopaedics, Rheumatology and Musculoskeletal Science, University of Oxford, Oxford, UK; 6 Department of Orthopaedic Surgery, Haukeland University Hospital, Bergen, Norway; 7 Department of Clinical Medicine, University of Bergen, Bergen, Norway

## Abstract

Femoral neck fractures (FNFs) are associated with loss of function in all ages and excess mortality. The societal costs are high. Treatment needs to be tailored based on fracture type, functional demand, and physiological age of the patient. Internal fixation is often preferred for undisplaced FNFs and for displaced FNFs in young patients. Anatomical reduction is essential, but slight valgus is accepted. For a majority of those with displaced FNFs, a cemented hemiarthroplasty is the best alternative. This educational article suggests a treatment algorithm for FNFs and describes the evidence base for the recommended surgical techniques. Basicervical fractures, stress and pathological fractures are not included in this review.

## Demographics

Femoral neck fractures (FNFs) account for around 60% of hip fractures in patients aged 60–69 years. This proportion decreases to 45–50% in patients aged ≥80 years at the expense of multi-fragmentary trochanteric fractures [1]. Two-thirds of these fractures occur in women, though men are the majority among patients under 60 years. The mean age is around 80 years with at least 90% of fractures caused by low-energy injuries, most commonly a fall from standing height, and often occurring in the patient’s own home. High-energy trauma causes 0.6% of the hip fractures in those above 60 years, and 11% in patients younger than 60 [2].

Mortality in patients over 65 years with hip fracture is around 15% at 3 months and 25% at 1 year, illustrating the frailty of these individuals [3]. Men (as compared with women), older and sicker patients have an even higher excess mortality after the fracture [4].

## Classification

**Figure 1:**
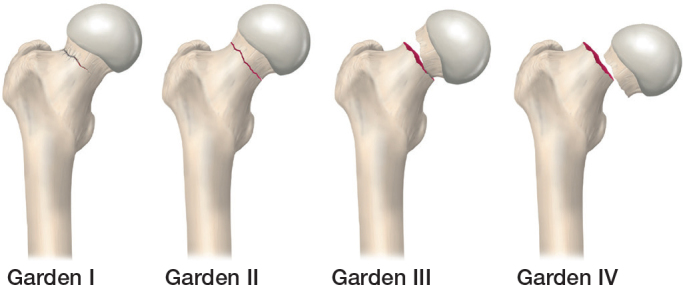
The Garden classification describing the displacement of the fracture based on the anteroposterior radiograph. Garden I is valgus-impacted, Garden II is the undisplaced fracture, Garden III is varus malaligned, and Garden IV is a displaced fracture (Illustration P Andersson).

FNFs are subjected to several classification systems. The commonly used Garden classification employs the anterior-posterior (AP) radiograph [5]. Garden I is valgus-impacted, Garden II is the undisplaced fracture, Garden III is varus malaligned, and Garden IV is a displaced fracture (Figure 1). For fractures treated with internal fixation, the risk of failure of fixation increases from Garden I (lowest risk) to Garden IV (highest risk) [5]. However, due to poor inter- and intra-rater reliability of the different grades of displacement, Garden I and II fractures are often grouped together as “undisplaced or minimally displaced” fractures, and Garden III and IV as “displaced” fractures [6].

**Figure 2:**
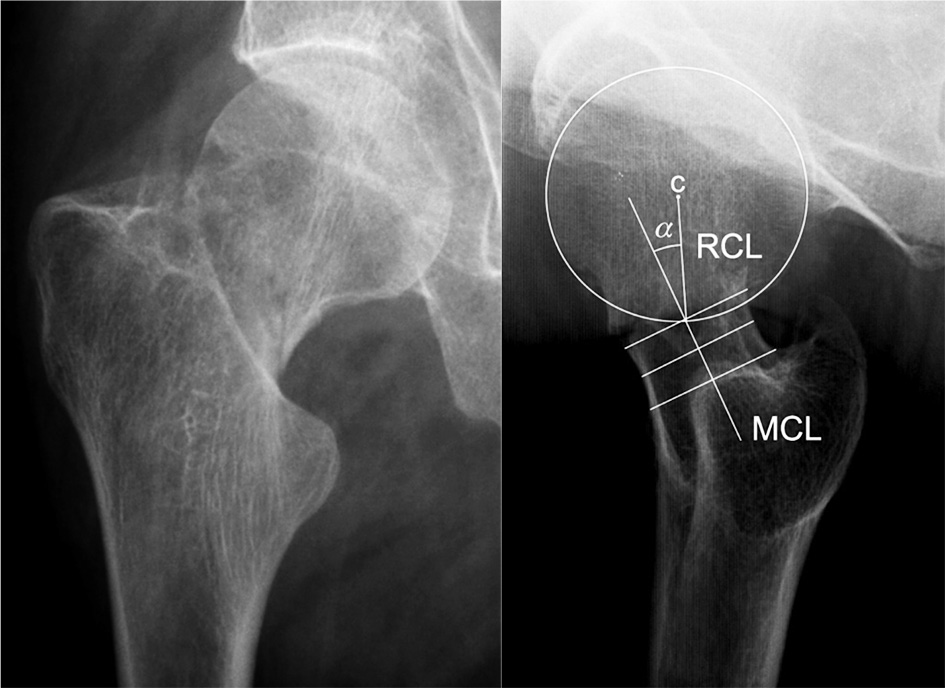
Preoperative anterior-posterior and lateral radiographs of undisplaced femoral neck fracture. The posterior tilt is measured as the angle (α) between mid-collum line (MCL), a line in the center of the femoral neck, and the radius collum line (RCL), a line from the center of the femoral head to the crossing of the femoral head circle and the MCL (Illustration and measurement method from Palm et al. [9]).

For the undisplaced or minimally displaced FNF the degree of posterior or anterior fracture tilt on lateral radiograph (Figure 2) has also been linked to an increased risk of fixation failure and healing disturbance [7,8]. Suggested cut-off values are 20° for posterior tilt and 10° for anterior [9].

**Take-home messages**

**Classification**

- Classifying femoral neck fractures (FNFs) into undisplaced or minimally displaced versus displaced fractures helps surgeons when deciding between fixation and arthroplasty.
- When considering fixation, the degree of tilt on the lateral radiograph should also be considered.

**Timing of surgery**

- Hip fracture surgery should be performed on the day of, or the day after, presentation to hospital.

**Treatment of undisplaced FNFs**

- Undisplaced FNFs should be treated with well-positioned internal fixation.
- Ongoing large randomized controlled trials (RCTs) will add information on the decision regarding fixation versus arthroplasty in older patients.

**Reduction of displaced FNFs**

- Reduce the fracture to (near) anatomical position on traction table with internal rotation, always avoiding a varus position.
- The surgeon needs to know more than 1 technique for closed reduction.
- If unsatisfactory fracture position after repeated attempts to reduce with different techniques, perform open reduction or convert to hip arthroplasty.

**Internal fixation of displaced FNFs**

- Avoid internal fixation for older patients with a displaced fracture who are medically fit to undergo an arthroplasty. Internal fixation leads to too many hip complications, reoperations, and worse patient-reported results.
- Internal fixation should be offered to those who are younger, healthy, and active, despite a high risk of reoperation.

**Arthroplasty for displaced FNFs**

- Hemiarthroplasty is the treatment of choice for most older patients with displaced FNF in order to reduce complications, dislocation in particular.
- Total hip arthroplasty (THA) can be considered if the older patient fulfills all 3 prerequisites: high activity level, no cognitive impairment, full walking ability (meaning outdoor walking without aids).
- THA is an option for patients with acetabular dysplasia, or symptomatic osteoarthritis, or rheumatoid arthritis in the injured hip.

**Techniques in hip arthroplasty for FNFs**

- Use a cemented femoral stem, preferably of anatomical or straight composite-beam design.
- The current literature supports the use of a direct lateral approach for arthroplasty in FNF to reduce the risk of dislocation.
- Consider a dual mobility cup for patients treated with THA if a posterior approach is used.

**Figure 3:**
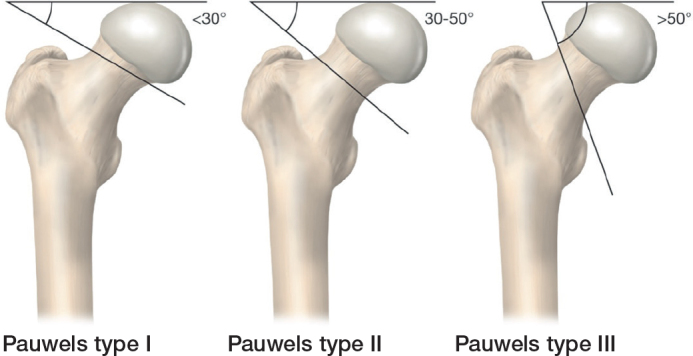
The Pauwels classification describing the shear angle of the fracture based on the anteroposterior radiograph [9] (Illustration P Andersson).

**Figure 4:**
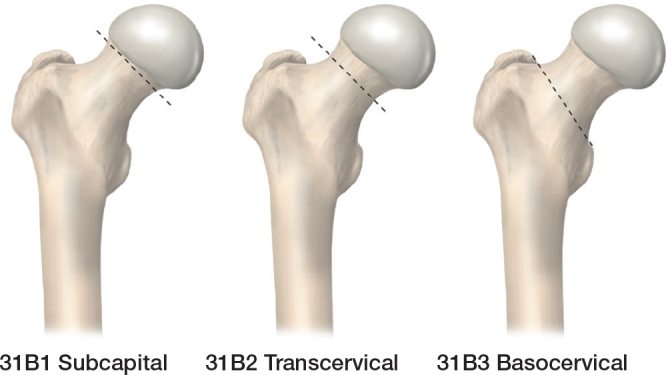
The 2018 AO/OTA system for classifying femoral neck fractures based on location [10] (Illustration P Andersson).

Other classifications are less commonly used, again due to issues related to observer reliability. These include the Pauwels classification, which is based on the AP radiograph and describes the shear angle of the fracture [10] (Figure 3). The 2018 AO/OTA system classifies FNFs in 3 levels, based on location (Figure 4), then displacement and finally shear angle, resulting in a total of 13 types of FNFs [11].

## Diagnostics

Plain calibrated pelvic radiographs and hip radiographs, AP and lateral views, are gold standard for imaging of a suspected hip fracture. On clinical suspicion of fracture and negative radiographs, an occult hip fracture can be diagnosed by magnetic resonance imaging (MRI). MRI has both high specificity and sensitivity compared with computed tomography (CT). CT carries a higher risk of false negative results but is more available [12].

## Timing of surgery

To avoid medical complications, such as infection, pressure ulcers, and delirium, patients with FNF should be prioritized for early surgery. Waiting time to surgery of more than 24 hours has been associated with increased 30-day mortality and medical complications [13]. Reducing waiting time is important for patients with severe comorbidity and high age [4]. A randomized controlled trial (RCT) comparing accelerated surgery within 6 hours with surgery within a median of 24 hours found no reduction of mortality or major complications [14]. Looking at the prognosis of the fracture itself, no relationship has been found between waiting time to surgery and avascular necrosis (AVN) following osteosynthesis [15]. For younger patients, no difference in the risk of non-union and AVN between surgery before and after 12 hours is found [16]. Thus, the fear of developing AVN or non-union should not be decisive regarding time to surgery. Sufficient experience of the surgeon and the team is more important, and surgical intervention can be scheduled during the daytime.

Several national evidence-based guidelines on treatment of hip fractures recommend surgery on the day of, or the day after, presentation to hospital [17-19]. There are few medical reasons that require surgery to be delayed beyond 48 hours. Use of direct-acting anticoagulants (DOACs) is one potential obstacle to early surgery [20], most likely due to fear of spinal hematoma. In these cases, surgery under general anesthesia can be performed without increasing mortality or incidence of postoperative delirium compared with spinal anesthesia [21]. When surgery is performed within the recommended time frame, other factors such as primary displacement, quality of reduction of the fracture, and the experience level of the surgeon are probably more important for the surgical result [22,23].

## Undisplaced FNF

For a “true” undisplaced FNF (Garden I or II with < 20° posterior tilt) (see Figure 1), the primary choice of treatment is internal fixation (IF) according to most guidelines [1,17,18]. Meta-analyses report reoperation rates 1 year after IF of around 10–12% [7], which correlates well with larger register studies demonstrating 6–11% 1-year reoperation rates [24-26]. The reoperation rates decrease with younger age and the conversion rate to arthroplasty within 2 years after primary IF is approximately 7% in those 50–59 years old and < 1% in patients below 50 years [27].

Fracture displacement and quality of reduction is the main reason for failure as described below for displaced FNF. In addition, the positioning of the implant is important. Minimizing the tip–apex distance (TAD) for sliding hip screws reduces the risk of cut-out in extracapsular fractures. We believe TAD results are transferable to FNFs. An implant positioning (IMPO) score for screws or pins has been developed for undisplaced FNF [28]: screws or pins should be placed close to the inferior calcar and postero-superior cortex to achieve maximum rotational stability. Screws should be parallel to permit compression when weightbearing. Screw tips should be anchored in bone of good strength, meaning a short tip–head distance. Finally, the implant should neither be placed in the inferior 15 mm of the femoral head (implants in varus) nor in the anterior 1/4 and superior 15 mm of the femoral head (increased risk of cut-out). The IMPO score might be transferable to displaced FNF as well.

Hemiarthroplasty is proposed as an alternative to IF for undisplaced FNF in older patients due to lower implant-related complication rate and lower reoperation rate [26,29,30]. 3 large-scale RCTs are currently being conducted that will provide more evidence [31-33].

## Displaced FNF

### Reduction and internal fixation

The outcome of IF is highly influenced by fracture displacement, and less by the choice of implant, with reduction being the most important risk factor for failure [23,34]. Anatomical reduction is mandatory to optimize healing conditions and implant positioning. If anatomical reduction is not possible, slight valgus and dorsal angulation is acceptable. Varus should be avoided.

Various closed reduction techniques for displaced FNFs have been described, in either neutral position or flexion of the hip joint [35]. Most of them are performed on a fracture table with traction of the injured limb. Any manipulation of the fractures is done by applying manual force such as traction, internal rotation, or pressure on the femoral fragment. Major displacement may warrant the aid of traction and fine-tuning with semi-open reduction with retractors or joysticks.

If closed reduction with traction is unsuccessful, the youngest patients may benefit from open reduction performed by a surgeon with experience in open reduction techniques. The Watson-Jones approach enables open reduction and fixation through the same window. To achieve better visualization of the fracture the more anterior Smith–Petersen approach gives a direct approach to the fracture. For fixation, an extra lateral incision may be necessary for implant positioning. A middle-aged patient can be treated with an arthroplasty immediately if closed reduction is unachievable. As always, different treatment options must have been discussed with the patient before surgery.

Cannulated screws have similar reoperation rates to sliding hip screw [36,37]. For the Pauwels 3 vertical and transcervical fracture type (see Figure 3), sliding hip devices could have biomechanical advantages [37,38].

### Arthroplasty

*Stem types and bone cement.* Both fixation and stem type can influence the outcome after hemiarthroplasty for FNF. A cemented arthroplasty reduces the risk of reoperation, in particular due to periprosthetic femoral fracture, compared with an uncemented arthroplasty [39-42]. Bone cement has also been found to modestly increase health-related quality of life [41]. No difference in mortality between cemented and uncemented hemiarthroplasties has been reported with longer follow-up [42-44]. Nevertheless, use of bone cement is associated with increased mortality in the first 1–2 postoperative days compared with uncemented arthroplasties [43,44]. This may be related to bone–cement implantation syndrome (BCIS) characterized by hypoxia, hypotension, and loss of consciousness. Severe BCIS is associated with an increased risk of perioperative death, and vigilance when operating on high-risk patients (ASA 3 and 4, cardiopulmonary disease) is important [45]. In order to reduce the risk of BCIS it is recommended to carefully prepare, wash, and dry the femoral canal, to avoid excessive pressure during cementation, and to cooperate and communicate closely with the anesthesiologist around the time of cementation [46]. Antibiotic-loaded bone cement is preferred to reduce risk of infection [47].

Regarding stem type, large studies have reported higher risk of periprosthetic fracture when using a collarless polished taper-slip stem compared with anatomic and straight composite-beam stems in both total hip arthroplasty (THA) and hemiarthroplasties [48-50].

*Bearings—bipolar, unipolar, dual mobility cup.* A hemiarthroplasty head can articulate against the acetabulum (unipolar head) or have an additional inner bearing (bipolar head). Underpinned by biomechanical studies, the bipolar head has been thought to carry a lower risk of acetabular erosion. However, when clinical trials are summarized, no difference of clinical relevance is detected [51].

THA with a dual mobility cup (DMC) may be used for patients who fill prerequisites for a primary THA but also are at risk of dislocation. In combination with a posterior approach a DMC can reduce the risk of dislocation, whilst it is more unclear whether there are any benefits when combined with other types of approaches [52]. The DUALITY RCT is closed and will provide information on DMC versus conventional THA [53].

*Surgical approach.* The most common approaches to the hip when performing arthroplasty are posterior and direct lateral approaches. Other anterolateral and anterior types of exposure can also be used. Register studies have demonstrated that the direct lateral approach has significantly lower revision risk due to dislocations than the posterior approach [39,52,54,55]. When comparing the direct lateral with the posterior approach for hemiarthroplasty, an RCT including 555 patients demonstrated a 5.5% dislocation rate with a posterior approach compared with 0.4% with a lateral [56]. Another RCT could not demonstrate any difference between the 2 approaches in 216 patients and had low dislocation rates in both groups [57]. Notably, this RCT studied a piriformis-preserving posterior approach, not a standard posterior approach. This enhancement of the posterior approach has demonstrated low dislocation rates in another RCT [58] and in cohort studies [59,60].

There have been concerns regarding the risk of abductor insufficiency and Trendelenburg gait due to splitting and partial detachment of the anterior part of gluteus medius in the direct lateral approach. Any association with patient-reported outcome is unclear as none of the approaches demonstrate superior hip function [61]. In a register study, less pain, better patient satisfaction, and better health-related quality of life was reported from those who had a posterior approach, compared with the group with a lateral approach [55]. However, only half of the patients responded to the questionnaires.

The lateral approach has a lower dislocation rate, and similar patient-reported outcome and functional outcomes compared with the standard posterior approach. It is therefore recommended in several national guidelines [17-19]. A posterior approach that spares the small external rotators could be an alternative, but more evidence is needed.

### Treatment overview

For patients with displaced FNF, the choice is mainly between reduction and IF or arthroplasty. Depending on the patient’s age, activity level, and coexisting diseases, the treatment methods have different advantages and disadvantages.

IF means that the patient keeps his/her native femoral head. It will also mean shorter surgical time, less soft tissue trauma, and less bleeding. The disadvantage is that the fracture may affect blood circulation to the femoral head, which leads to a relatively high risk of non-union, AVN, mechanical failure, and reoperation [62].

**Figure 5:**
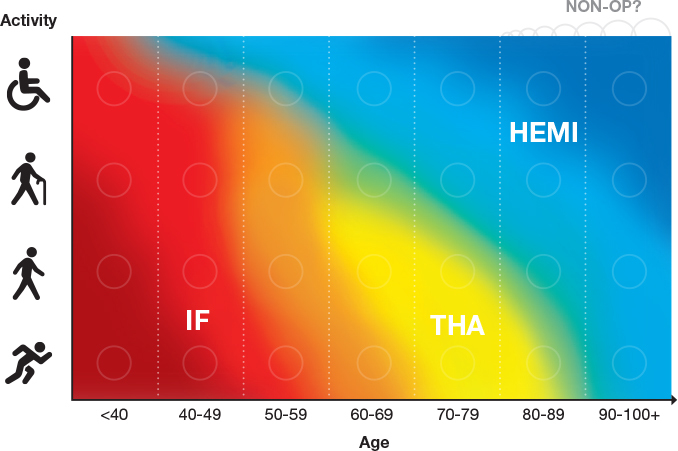
Patients and surgeons should consider both age and activity level when deciding on the surgical method of displaced FNFs. In areas where the methods overlap, the skills of the surgeon and of the team can also influence the choice, and shared decision-making is advocated when possible (Illustration P Andersson).

Arthroplasty is more extensive, but the overall risk of hip complications and reoperation is lower than after IF. Patients who have had a primary arthroplasty usually have less pain and better function than those who have received IF [62]. Figure 5 gives a schematic view of how different surgical methods may be preferred regarding the patient’s age and activity level. Note that the highest activity level equals the highest score in an activity assessment suitable for older individuals, meaning regular and hard exercise several times a week with strenuous physical exertion. To use an activity assessment score [63] may be helpful.

### Choice between internal fixation and arthroplasty

Those who are of working age, healthy, and physically active are usually treated with IF. A long-expected survival after the fracture increases the risk of late arthroplasty complications such as aseptic loosening. IF is therefore believed to be a better choice, even though one-third will need secondary surgery within a 5-year period [27,64]. In such cases, a younger individual will have sufficient capacity to cope with 2 procedures, IF and secondarily a hip arthroplasty, when needed without losing too much of their functional capacity. In cases where IF is considered a suitable treatment, information concerning the risk of reoperation must be given and the patient should be followed up until healing occurs. If secondary treatment with conversion to arthroplasty is needed, prompt surgery should be provided, to minimize the period of discomfort and immobilization.

Those who have a reduced activity level, due to aging or comorbidities, also have a poorer capacity to cope with repeated surgical interventions. This group also has slightly more local complications after IF than younger individuals. In the short to medium term, arthroplasty causes less pain and better function than IF. Therefore, arthroplasty is the preferred treatment for individuals with a limited lifespan (51).

### Choice between THA and hemiarthroplasty

For the majority of those who sustain a FNF, the choice of THA or hemiarthroplasty will not affect the clinical course, at least not during the first 2 years [51]. A hemiarthroplasty is a slightly less extensive intervention and carries a lower risk of dislocation [51]. The disadvantage is the potential risk of acetabulum erosion, which can lead to pain and poorer function. Hemiarthroplasties are the first choice for older frail patients, patients with cognitive impairment (to decrease the risk of dislocation), and patients with a lower functional level [65].

The somewhat greater surgical trauma in THA has been accepted in the light of theoretically better hip function over time. THA has therefore been used for healthier, slightly younger individuals with displaced FNF. Usually, walking outside the home without aids and normal cognitive ability have been used as an indication for THA. It has been assumed that a high degree of activity increases the risk of acetabular erosion after hemiarthroplasty, but there is no short-term difference between the surgical methods [66]. In patients with symptomatic osteoarthritis or arthritis in the injured hip, a THA is advantageous. Acetabular dysplasia is a risk factor for dislocation of hemiarthroplasty, and such radiological findings can speak in favor of THA [67].

## Special considerations

### Patients who are permanently immobile or in end-stage of life

When the patient is either in an acute life-threatening stage, or completely unable to stand up, other treatment methods can be considered and discussed. The individual’s own wishes and presumed benefits should guide the choice of treatment [68]. A minimal intervention to stabilize the fracture can relieve the pain. In other cases, palliative care and nonoperative treatment should be considered. Resection arthroplasty, i.e., Girdlestone procedure, should in principle never be used as emergency treatment [69]. High treatment satisfaction after nonoperative treatment, and even non-hospitalization, in a selective population with hip fractures has been reported but further research in this area is needed [68].

## Rehabilitation

This review does not cover the general aspects of early weight-bearing mobilization and continuous rehabilitation after hip fracture. Movement restrictions and mandatory aids are not needed after arthroplasty in fracture patients, at least not when the direct lateral approach is used [70]. The younger the patient and the more displaced or comminuted the fracture, expert opinions have promoted protected weightbearing after IF in the postoperative phase. No clinical study has been able to show any advantage of such a regime [71], and weight-bearing restrictions may hamper the rehabilitation process [72] and may add to fear of movement. Besides, most older patients cannot adhere to weight-bearing restrictions [73].

## Funding, use of AI, and disclosures

No specific funding was sought for this article. AI tools were not used. Authors report the following disclosures: Honoraria: OW—Link Sweden, Smith & Nephew, Depuy, Swemac; JEG—Link Norway, Smith & Nephew, DepuySynthes, Heraeus Medical; and CR—Link Sweden, Swemac. Participation on Boards: OW, SM. Leadership or fiduciary role: OW—Director Swedish Fracture Register, Chair Swedish Orthopaedic Trauma Society. Grants: MC—National Institute for Health Research (HTA) FRUTI – Fix or Replace Undisplaced Intracapsular fractures Trial of Interventions; BV—Swemac.Complete disclosure of interest forms according to ICMJE are available on the article page, doi: 10.2340/17453674.2025.44354

## Notes

All authors contributed equally to the review of the literature, drafting of the manuscript, and the final version of it.

Handling co-editors: Li Felländer-Tsai and Bart A Swierstra.

Acta thanks Christina Frölich Frandsen and JLC van Susante for help with peer review of this manuscript.
